# The causal relationship between gut microbiota and COVID-19: A two-sample Mendelian randomization analysis

**DOI:** 10.1097/MD.0000000000036493

**Published:** 2023-02-02

**Authors:** Siyu Tian, Wenhui Huang

**Affiliations:** a Proctology Department, School of Clinical Medicine, Chengdu University of Traditional Chinese Medicine, Chengdu, China; b Cardiothoracic Surgery Department, Hospital of Chengdu University of Traditional Chinese Medicine, Chengdu, China.

**Keywords:** COVID-19, gut microbiota, Mendelian randomization analysis, the causal relationship

## Abstract

Recent studies have shown that gut microbiota is associated with coronavirus disease 2019 (COVID-19). However, the causal impact of the gut microbiota on COVID-19 remains unclear. We performed a bidirectional Mendelian randomization. The summary statistics on the gut microbiota from the MiBioGen consortium. Summary statistics for COVID-19 were obtained from the 6th round of the COVID-19 Host Genetics Initiative genome-wide association study meta-analysis. Inverse variance weighting was used as the main method to test the causal relationship between gut microbiota and COVID-19. Reverse Mendelian randomization analysis was performed. Mendelian randomization analysis showed that *Intestinimas.id.2062* was associated with an increased risk of severe COVID-19. *Bifidobacterium.id.436, LachnospiraceaeUCG010.id.11330, RikenellaceaeRC9gutgroup.id.11191* increase the risk of hospitalized COVID-19. *RuminococcaceaeUCG014.id.11371* shows the positive protection on hospitalized COVID-19. There is no causal relationship between gut microbiota and infection with COVID-19. According to the results of reverse Mendelian randomization analysis, no significant causal effect of COVID-19 on gut microbiota was found. The study found that gut microbiota with COVID-19 has a causal relationship. This study provides a basis for the theory of the gut-lung axis. Further randomized controlled trials are needed to clarify the protective effect of probiotics against COVID-19 and the specific protective mechanisms. This study has important implications for gut microbiota as a nondrug intervention for COVID-19.

## 1. Introduction

Coronavirus disease 2019 (COVID-19), caused by severe acute respiratory syndrome coronavirus 2 (SARS-CoV-2), has had a devastating impact on humans worldwide.^[1,2]^ As of April 9, 2023, more than 762 million confirmed cases and more than 6.8 million deaths had been reported globally.^[3]^ Due to the alarming spread of SARS-CoV-2 around the world, the World Health Organization declared a global COVID-19 epidemic on March 11, 2020.^[4–6]^ A large-scale study has shown that nondrug interventions have a significant impact on both COVID-19 mobility and pandemic indicators, especially at the beginning and most severe stages of an emergency.^[7]^ Some government agencies have resorted to nondrug interventions such as international travel restrictions, service restrictions, stay-at-home, school closures, and reduced events and gatherings to limit the spread and prevalence of COVID-19.^[8]^ In addition to the above nondrug interventions, we need more nondrug interventions to stop the spread and epidemic of COVID-19.

The human gut microbiome consists of 10^14^ resident microbial species, including bacteria, archaea, viruses, and fungi.^[9]^ The gut bacteria of healthy individuals are mainly composed of 4 phyla: *Actinobacteria, Firmicutes, Proteobacteria*, and *Bacteroidetes*.^[10]^ Interestingly, the gut microbiota has been shown to influence lung health through an important link between the gut microbiota and the lungs, known as the “gut-lung axis.”^[11]^ The gut-lung axis is thought to be bidirectional, which means that when inflammation occurs in the lungs, the gut microbiota can be affected, and gut microbial metabolites can also affect the lungs through the blood.^[12]^ Based on this theory, scholars have raised the intriguing possibility that COVID-19 may also have an impact on the gut microbiome.^[13]^ Recent studies have shown that COVID-19 seriously affects the gastrointestinal tract.^[14–18]^ SARS-CoV-2 virus can be detected in the stool samples of patients with COVID-19.^[19–21]^ Patients with COVID-19 are usually accompanied by gastrointestinal symptoms.^[22,23]^ Large amounts of replicating SARS-CoV-2 virus have been observed in small and large bowel biopsies from patients with COVID-19, mainly in intestinal epithelial cells.^[24]^

However, direct evidence for the causal influence of gut microbiota on COVID-19 susceptibility, severity, and hospitalization characteristics is lacking. Most previous traditional studies have been case-control studies. However, case-control studies on the relationship between gut microbiota and COVID-19 are susceptible to the influence of factors such as age, environment, dietary pattern, and lifestyle, which may easily lead to biased results.^[25]^ It is difficult to control these confounding factors in observational studies, and a large number of human resources and time-consuming follow-up are required in randomized controlled studies, which limits the inference of causal relationships between gut microbiota and COVID-19. Mendelian randomization (MR), which uses a genetic variation to construct instrumental variables (IVs) of exposure, is increasingly being applied to infer credible causal relationships between risk factors and disease outcomes.^[26]^ Since offspring genes are randomly assigned by parents at conception, the association between genetic variants and outcomes is not affected by common confounding factors, and MR analysis can effectively rule out confounding factors and determine reasonable causal relationships.^[27]^ MR has been widely used to explore the causal relationship between intestinal microbiota and diseases, including metabolic diseases,^[28]^ autoimmune diseases,^[29]^, etc. Given the uncertainty of the causal relationship between gut microbiota and COVID-19, we used large-scale genome-wide association study (GWAS) data to evaluate the potential causal effects of gut microbiota on COVID-19 susceptibility, hospitalized COVID-19 patients, and severe COVID-19 patients using an MR analysis. In summary, this study evaluated the influence of gut microbiota on COVID-19 and attempted to provide constructive suggestions for the prevention and intervention of COVID-19 through gut microbiota and reliable evidence for the theory of the “gut-lung axis.”

## 2. Methods

### 2.1. Study design

We conducted a total of 2 MR analyses using pooled statistics from a GWAS study to investigate the bidirectional association between COVID-19 and gut microbiota. Forward MR analysis uses gut microbiota as exposure and COVID-19 as outcome, while reverse MR uses COVID-19 as exposure and gut microbiota as outcome. The MR analysis needs to satisfy 3 core assumptions: namely, the genetic variation must be associated with the gut microbiota, independent of confounders associated with the association between the gut microbiota and COVID-19, and associated with COVID-19 only by association with the gut microbiota.

### 2.2. Data source

Gut microbiota data were derived from the largest genome-wide meta-analysis of gut microbiota composition published to date by the MiBioGen consortium.^[30,31]^ The MiBioGen consortium study curated and analyzed genome-wide genotype and 16S fecal microbiome data from 18,340 individuals (24 cohorts). Most of these cohorts were from Europe (n = 13,266), and most cohorts included adults or adolescents (n = 16,632). The resulting categories were sorted using direct classification binning. The MiBioGen consortium performed microbiota quantitative trait locus mapping analysis to identify host genetic variation that corresponds to genetic loci associated with the abundance level of bacterial taxa in the gut microbiota. The genus was the lowest taxonomic level in this study, and 131 genera were identified with an average abundance >1%, including 12 unknown genera.^[30]^ Therefore, after the removal of 12 unknown genera, 119 genera were included in this study for MR analysis.

Summary statistics of COVID-19 phenotypes were extracted from the 6th round of the COVID-19 Host Genetics Initiative GWAS meta-analysis in June 2021.^[32]^ A total of 2,586,691 Europeans participated in the study. COVID-19 infection was diagnosed as a positive SARS-CoV-2 infection based on laboratory testing (e.g., RNA, serologic tests), as reported by electronic health records, or as self-reported. The susceptibility phenotype of COVID-19 is to compare the COVID-19 patients with the control group of people not infected with COVID-19 (Ncase = 112,612, Ncontrol = 2,474,079). The hospitalization phenotype of COVID-19 is a comparison between hospitalized patients with COVID-19 and people without COVID-19 (Ncase = 24,274, Ncontrol = 2,061,529). The severe phenotype of COVID-19 is to compare the hospitalized patients who died of COVID-19 or needed ventilator support with people without severe COVID-19 or people infected with COVID-19 (Ncase = 8779, Ncontrol = 1,001,875).

### 2.3. Selection of instrumental variables

Single nucleotide polymorphisms (SNPs) with a genome-wide significance level (*P* < 1.0 × 10^−5^) were selected.^[33]^ The linkage disequilibrium between SNPs was calculated, and SNPs were clustered under the criteria of *R*^2^ ≥ 0.001 and window size of 10,000 kb, and the SNPs with the smallest value were retained. Allele frequency information was used to infer the leading strand allele when palindromic SNP was present.

### 2.4. Statistical analysis

All statistical analyses were performed with the use of R (version 4.2.2). MR analysis was also performed using the 2 sample MR package (version 0.5.6).^[34]^ This study used multiple methods, including inverse variance weighting (IVW), MR-Egger regression, weighted median, weighted model, and sample mode to verify whether there is a causal relationship between gut microbiota and COVID-19. We used the IVW for the primary analysis, which combined the Wald ratio for each SNP on the outcome to obtain an overall estimate of the causal effect of gut microbiota on COVID-19.^[35]^ In addition, additional MR analyses, such as MR-Egger regression, weighted median, weighted model, and sample mode, complement IVW to provide more robust causality estimates.^[36]^ MR-Egger regression can test for both horizontal pleiotropy and heterogeneity tests and if the intercept term is equal to zero, this indicates that horizontal pleiotropy is not present and the results of MR-Egger regression are consistent with IVW.^[37]^ To assess the causal relationship between gut microbiota and COVID-19, we also performed reverse MR analysis on bacteria that were found to have a causal relationship with COVID-19 in the forward MR analysis. The methods and settings used were consistent with forward MR.

Sensitivity analysis was performed using the Cochran *Q* test, Egger-intercept test, and leave-one-out analysis. Cochran *Q* test statistics were used to quantify heterogeneity in IVs. The Egger-intercept test was used to test for horizontal pleiotropy in the studies. In addition, to identify potentially heterogeneous SNPS, a leave-one-out analysis was performed by omitting each SNP in turn. The single IV strength of gut microbiota was assessed by calculating the F-statistic using the formula *F* =  *β*^2^_exposure_/SE^2^_exposure_. No significant weak instrument bias was considered if the corresponding *F*-value was >10.^[38]^

## 3. Results

**Figure 1.**
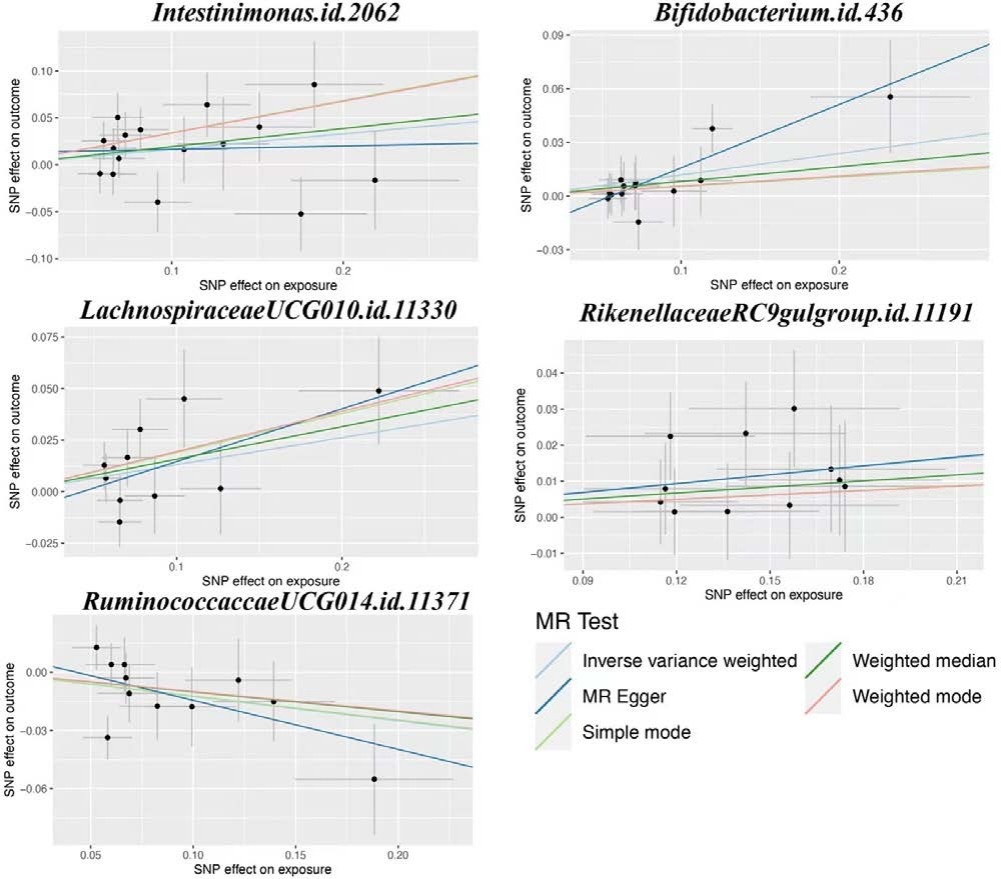
The MR analysis of the positive gut microbiota and COVID-19. COVID-19 = coronavirus disease 2019, MR = Mendelian randomization, SNP = single nucleotide polymorphism.

**Figure 2.**
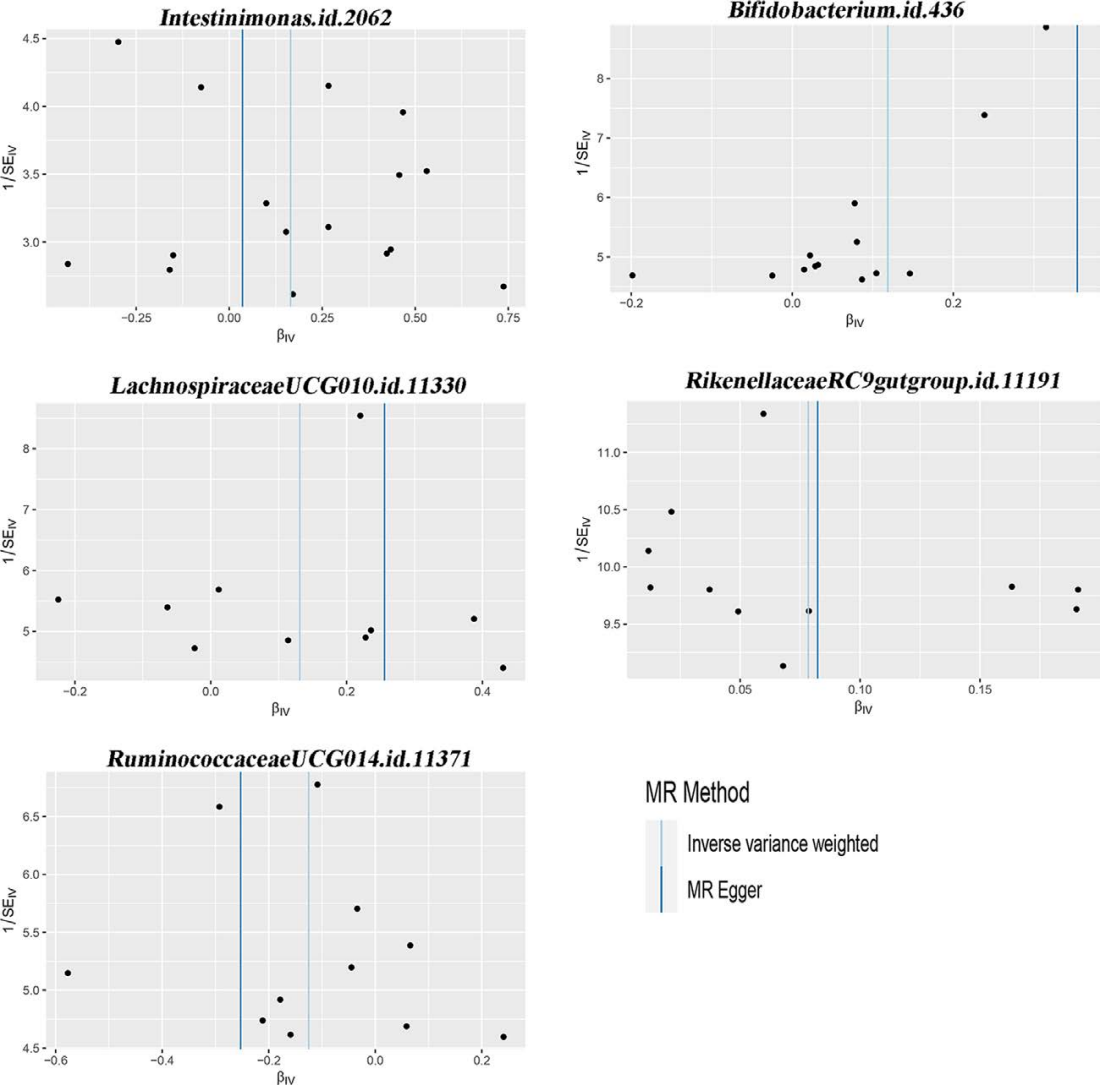
The scatter plots of the positive gut microbiota and COVID-19. COVID-19 = coronavirus disease 2019, MR = Mendelian randomization.

**Figure 3.**
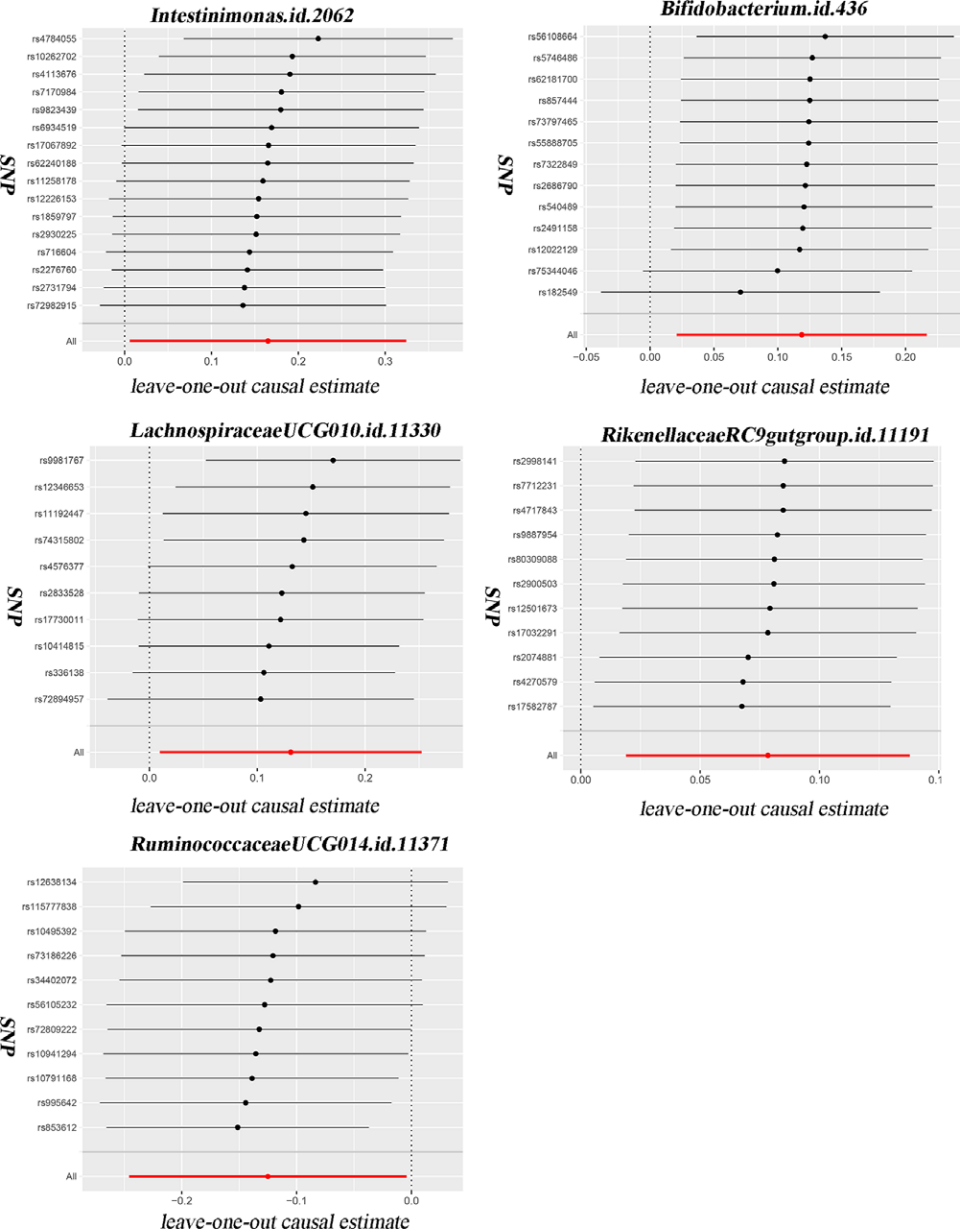
The leave-one-out analysis plots of positive gut microbiota and COVID-19. COVID-19 = coronavirus disease 2019, SNP = single nucleotide polymorphism.

This study will verify the causal relationship between gut microbiota and infection with COVID-19, hospitalized COVID-19, and severe COVID-19. According to the IVs selection criteria, a total of 6410 SNPs were used as IVs for 119 gut bacterial genera. Details of positive IVs are provided in the additional file: Table S1, Supplemental Digital Content, http://links.lww.com/MD/L311. Additional file: Table S2, Supplemental Digital Content, http://links.lww.com/MD/L312, shows the MR analysis of 119 gut microbiota and infection with COVID-19, hospitalized COVID-19, and severe COVID-19, respectively. Figure 1 illustrates the MR analysis of the positive gut microbiota and COVID-19. Figure 2 shows the scatter plots of the positive gut microbiota and COVID-19. Figure 3 illustrates the leave-one-out analysis plots of positive gut microbiota and COVID-19.

MR analysis of gut microbiota and severe COVID-19 is shown in Table 1. *Intestinimonas.id.2062* is causally related to severe COVID-19, and increases the risk of severe COVID-19 (*P* = .042, OR = 1.179, 95% CI = 1.006–1.383). The *P* value of the Cochran *Q* test was 0.271 > 0.05, indicating that there was no heterogeneity. The *P* value of the Egger-intercept test was 0.516 > 0.05, indicating that there was no horizontal pleiotropy. Leave-one-out analysis showed that all SNP’s All value >0, which proved the reliability of the result.

**Table 1:** Positive results of MR analysis of gut microbiota and COVID-19.

Bacterial genus (exposure)	MR method	Number of SNPs	OR	95% CI	*P*-value	The Cochran Q test’ *P*-value	Egger-intercept test’ *P*-value
*Intestinimonas.id.2062*	MR-Egger	16	1.036	0.685–1.567	.868		
	Weighted median	16	1.213	0.976–1.507	.081		
	Inverse variance weighted	16	1.179	1.005–1.383	.042	.271	.516
	Simple mode	16	1.409	0.938–2.115	.118		
	Weighted mode	16	1.403	0.909–2.164	.146		
*Bifidobacterium.id.436*	MR-Egger	13	1.424	1.087–1.865	.025		
	Weighted median	13	1.085	0.952–1.236	.219		
	Inverse variance weighted	13	1.126	1.021–1.241	.017	.824	.093
	Simple mode	13	1.054	0.829–1.341	.672		
	Weighted mode	13	1.057	0.839–1.332	.642		
*LachnospiraceaeUCG010.id.11330*	MR-Egger	10	1.291	0.937–1.778	.155		
	Weighted median	10	1.171	0.996–1.376	.055		
	Inverse variance weighted	10	1.139	1.009–1.287	.034	.306	.431
	Simple mode	10	1.208	0.911–1.603	.221		
	Weighted mode	10	1.215	0.946–1.559	.160		
*RikenellaceaeRC9gutgroup.id.11191*	MR-Egger	11	1.085	0.745–1.581	.678		
	Weighted median	11	1.057	0.977–1.144	.164		
	Inverse variance weighted	11	1.081	1.019–1.147	.009	.916	.983
	Simple mode	11	1.042	0.927–1.171	.500		
	Weighted mode	11	1.041	0.926–1.171	.507		
*RuminococcaceaeUCG014.id.11371*	MR-Egger	11	0.776	0.551–1.095	.183		
	Weighted median	11	0.903	0.778–1.048	.181		
	Inverse variance weighted	11	0.882	0.782–0.995	.042	.286	.455
	Simple mode	11	0.884	0.694–1.126	.344		
	Weighted mode	11	0.905	0.720–1.138	.415		

CI = confidence interval, COVID-19 = coronavirus disease 2019, MR = Mendelian randomization, OR = odds ratio, SNP = single nucleotide polymorphism.

The MR analysis of gut microbiota and hospitalized COVID-19 is shown in Table 1. The genus *Bifidobacterium.id.436* shows a causal relationship with hospitalized COVID-19, and increases the risk of hospitalized COVID-19 (*P* = .017, OR = 1.126, 95%CI = 1.021–1.242). The Cochran *Q* test’ *P* = .824 > 0.05, indicating that no heterogeneity is present. The Egger-intercept test’ *P* = .093 > 0.05, shows that there is no horizontal pleiotropy. Leave-one-out analysis indicated that all SNPs’s All value >0, which proved the result is reliable. *LachnospiraceaeUCG010.id.11330* genus shows a causal relationship with hospitalized COVID-19, which is a risk factor for hospitalized COVID-19 (*P* = .034, OR = 1.139, 95% CI = 1.009–1.287). The Cochran *Q* test’ *P* = .306 > 0.05, which suggests that there is no heterogeneity in the studies. The Egger-intercept test’ *P* = .431 > 0.05, indicating that there is no horizontal pleiotropy. Leave-one-out analysis shows that all SNPs’s All value >0, proving the result is reliable. *RikenellaceaeRC9gutgroup.id.11191* bacteria suggests that there is a causal relationship between hospitalized COVID-19 and the *Rikenellaceaerc9gutgroup.id.11191* bacteria may increase the risk of hospitalized COVID-19 (*P* = .009, OR = 1.081, 95% CI = 1.019–1.147). The Cochran *Q* test’ *P* = .916 > .05, suggesting that there is no heterogeneity in the studies. The Egger-intercept test’ *P* = .983 > .05, indicating that there is no horizontal pleiotropy in the studies. Leave-one-out analysis shows that all SNPs’s All value >0, proving the reliability of the result. *RuminococcaceaeUCG014.id.11371* has been shown to have a causal relationship with hospitalized COVID-19, which has a protective effect on hospitalized COVID-19 (*P* = .042, OR = 0.822, 95% CI = 0.782–0.995). The Cochran *Q* test’ *P* = .286 > .05, suggesting that there is no heterogeneity and bias present in the studies. The Egger-intercept test’ *P* = .455 > .05, showing that there is no horizontal pleiotropy. Leave-one-out analysis shows that all SNPs’s All value >0, demonstrating the reliability of the result.

The MR analysis of gut microbiota and infection with COVID-19 is shown in Table 1, and there is a causal relationship between the absence of gut microbiota and patients with COVID-19.

Among the 5 causal associations, F-statistics for individual IVs ranged from 18.382 to 88.429, eliminating the bias of weak IVs.

Results were analyzed according to reverse MR. As shown in Table 2, the results of MR analysis of COVID-19 as the exposure factor and 5 gut microbiota as the outcome can obtain all *P* values of IVW >0.05, which means that COVID-19 will not cause changes in these 5 gut microbiota.

**Table 2:** Reverse MR of 5 positive gut microbiota and COVID-19.

Bacterial genus (outcome)	MR method	Number of SNPs	*P*-value
*Intestinimonas.id.2062*	MR-Egger	40	.955
	Weighted median	40	.979
	Inverse variance weighted	40	.858
	Simple mode	40	.941
	Weighted mode	40	.879
*Bifidobacterium.id.436*	MR-Egger	42	.543
	Weighted median	42	.838
	Inverse variance weighted	42	.420
	Simple mode	42	.267
	Weighted mode	42	.872
*LachnospiraceaeUCG010.id.11330*	MR-Egger	42	.111
	Weighted median	42	.156
	Inverse variance weighted	42	.951
	Simple mode	42	.671
	Weighted mode	42	.222
*RikenellaceaeRC9gutgroup.id.11191*	MR-Egger	39	.837
	Weighted median	39	.837
	Inverse variance weighted	39	.978
	Simple mode	39	.912
	Weighted mode	39	.801
*RuminococcaceaeUCG014.id.11371*	MR-Egger	42	.911
	Weighted median	42	.677
	Inverse variance weighted	42	.754
	Simple mode	42	.803
	Weighted mode	42	.741

COVID-19 = coronavirus disease 2019, MR = Mendelian randomization, SNP = single nucleotide polymorphism.

## 4. Discussion

In order to slow the spread of COVID-19 and future outbreaks, studies have shown that lifting restrictions imposed by COVID-19 at the rate of vaccination progress can effectively control the pandemic and keep morbidity low.^[39]^ Meanwhile, research shows that there is an urgent need to reduce the number of people infected with COVID-19 and take appropriate isolation measures; protecting children by developing and approving anti-COVID-19 medicines for children; mass vaccination of the population.^[40]^ Gut microbiota is also worthy of further study as a nondrug intervention measure for COVID-19. In this study, we performed a 2-sample MR analysis to assess the causal relationship between gut microbiota and COVID-19. We found *RuminococcaceaeUCG014.id.11371* has a protective effect on hospitalized COVID-19. At the same time, some gut microbiota has been found to be potential risk factors for severe or hospitalized COVID-19.

Many observational studies have reported associations between gut microbiota and COVID-19.^[41–43]^ *RuminococcaceaeUCG014.id.11371* was found to be associated with a lower risk of COVID-19, which was consistent with our study results.^[44,45]^ The relevance of gut microbiota was realized when viral RNA was detected in the feces of 50% of hospitalized COVID-19 patients in Italy, characterized by the absence of gut microbiota in symbionts such as *Bacteriaceae, Lactococcaceae*, and *Ruminococcaceae*, which were replaced by *Enterococcus, Staphylococcus, Serratia*, and *Klebsiella*, as well as *Lactobacillus, Lactococcus, Actinomyces*, etc.^[46]^ In patients infected with COVID-19, depletion of *Bacteriaceae, Ruminococcaceae*, and *Lachnospiraceae* was observed.^[47]^ Because short-chain fatty acids have strong anti-inflammatory activity,^[48]^ they regulate the interferon (IFN) response to viral infection.^[49]^ Recent studies have shown that the content of short-chain fatty acids in *Ruminococcus* is increased in patients with less severe COVID-19,^[50]^ further indicating that *Ruminococcus* can reduce the risk of COVID-19. One study used stool samples from convalescent COVID-19 patients and acute COVID-19 patients and found that *Intestinimonas* numbers were reduced in convalescent COVID-19 patients, and *Intestinimonas* was negatively correlated with cough.^[51]^ It is consistent with our finding that *Intestinimonas* is associated with an increased risk of COVID-19. The study has found a strong inverse association between the abundance of a variety of beneficial bacteria and the development of postacute COVID-19 syndrome.^[52]^ Loss of several commensal bacteria, including the genera *Bifidobacterium, Roseburia*, and *Faecalibacteria*, which are known to have immunomodulatory functions, is particularly associated with persistent symptoms in patients who have recovered from COVID-19.^[50]^ The occurrence of severe COVID-19 was associated with the high abundance of 4 intestinal microorganisms (i.e., *Burkholderia contaminans, Bacteroides nordii, Bifidobacterium longum*, and *Blautia* sp. CAG 257).^[51]^ In addition, proteins of disease-related bacteria, such as *B longum*, can be detected in the blood samples of COVID-19 patients.^[51]^ At present, it is still controversial that whether *Bifidobacterium* can increase or reduce the risk of COVID-19. Different research conclusions may be due to small sample sizes, racial differences, and different study designs. The controversy of *Bifidobacterium* requires a larger sample, multicenter randomized controlled trials to further confirm its role in COVID-19. Compared with fecal samples with low or no SARS-CoV-2 infection characteristics, fecal samples with severe infection characteristics SARS-CoV-2 had a higher abundance of bacteria that produce short-chain fatty acids, Zuo et al reported.^[52]^ In patients infected with COVID-19, the abundance of *Larnospiraceae* was reduced.^[45]^ This finding is contrary to our MR analysis and warrants further clinical investigation. There are no reports on the *Rikenellaceae* genus and COVID-19. So far, no study has reported the relationship between *Rikenellaceae* and COVID-19. Therefore, more studies are needed to confirm the relationship between *Rikenellaceae* and SARS-CoV-2. In general, there are few observational studies on a single bacterial species, and the observational sample size is small, and different observational designs will lead to different conclusions. Large-scale multicenter randomized controlled trials and basic research are needed to confirm the detailed causal relationship between gut microbiota and COVID-19.

The gut-lung axis is a bidirectional interaction between the respiratory mucosa and the gut microbiota, which is considered to be related to the pathological immune response to SARS-CoV-2.^[19]^ The influence of the gut microbiota on systemic immunity, as well as on respiratory tract infections, has recently been demonstrated in mouse and human experiments.^[53,54]^ Studies have shown that the gut microbiota plays an important role in the response of the lung by regulating homeostasis as well as the immune response during viral infection.^[55,56]^ Notably, researchers have reported a role for gut microbes to exert antiviral immunity in the respiratory tract by affecting epithelial cells, alveolar macrophages, and dendritic cells, and simultaneously by altering cellular and humoral immune responses.^[57,58]^ The gut microbiota influences type I IFN receptor expression in respiratory epithelial cells, limiting viral replication through IFN-α and IFN-β.^[57]^ In addition, the gut microbiota is also involved in the activation of specific CD4^+^ and CD8^+^ T lymphocytes, participates in the stable expression of pro-IL-1b, pro-IL-18, and NLRP3, and plays an important role in the occurrence of inflammatory responses.^[56]^ Several studies have also reported that signals from the gut microbiota exert different effects on the lung mucosa, such as enhancing antiviral status in epithelial or immune cells and controlling viral replication at the onset of infection.^[55,57]^ Infection of intestinal epithelial cells with SARS-CoV-2 is known to cause dysbiosis of gut microbiota, intestinal inflammation, and gastrointestinal symptoms.^[59]^ At the same time, an inflammatory biological environment and epithelial injury induce angiotensin converting enzyme (ACE) 2 expression and increase the replication of SARS-CoV-2 in intestinal epithelial cells.^[60]^ The prolonged duration of gastrointestinal symptoms in patients with COVID-19 is associated with immune dysregulation and delayed clearance of SARS-CoV-2.^[61]^ SARS-CoV-2 enters the host through the ACE 2 receptor, which is highly expressed in the respiratory and gastrointestinal tract.^[59,62]^ ACE plays an important role in controlling intestinal inflammation and gut microbial ecology.^[63]^

We found that in the MR analysis of gut microbiota and COVID-19, the types of bacteria with a causal relationship between gut microbiota and hospitalized COVID-19 were the most, which may be related to the use of antibiotics in hospitalized patients. Antibiotics cause disruption of the gut microbiome.^[64]^ Evidence suggests that antibiotic use leads to further disruption of the gut microbiota in COVID-19-positive patients, which is exacerbated by antibiotic exposure, but the effects generally do not persist after recovery.^[65]^ Therefore, for hospitalized patients with COVID-19, clinicians should pay attention to the disorder of gut microbiota and the aggravation of gastrointestinal symptoms caused by the use of antibiotics.

This study confirmed the causal relationship between gut microbiota and COVID-19. Therefore, treatment methods such as microbiota transplantation and probiotics can be used to improve the gut microbiota and reduce the gastrointestinal symptoms of COVID-19 patients, which is one of the methods to prevent COVID-19 with minimal impact on patients. Further studies in expanded longitudinal cohorts of COVID-19 patients with different severity levels will help to deepen the understanding of the role of the gut microbiota in COVID-19 disease progression and recovery. These findings may help to identify microbial targets and probiotic supplements to improve the treatment of COVID-19.

## 5. Strengths and limitations

Our study has several strengths. MR analysis excluded the interference of confounding factors and determined the causal relationship between intestinal microbiota and COVID-19. Genetic variation of gut microbiota was obtained from the largest GWAS meta-analysis available, ensuring instrument strength in the MR analysis. Cochran *Q* was used to test the heterogeneity of the studies, Egger intercept was used to test the horizontal pleiotropy of the studies, and Leave-one-out analysis was used to test the reliability of the results.

Our study also has some limitations. The lowest taxonomic level of exposure data was genus, which prevents us from further exploring the causal relationship between gut microbiota and COVID-19 at the species level. More genetic variants need to be used as IVs. The SNPS used in the analysis did not meet the traditional GWAS significance threshold (*P* < 5 × 10^−8^). To this end, we used false discovery rate correction to limit the likelihood of weak instruments. The sample size of gut microbiota was relatively small, so the results of reverse MR analysis may be affected by weak instrument bias, and reverse causality cannot be completely ruled out. Most of the participants in the GWAS gut microbiota data meta-analysis were of European descent, which may be confounded by ethnic stratification. Future MR studies on the causal relationship between gut microbiota and COVID-19 can be considered in European and non-European populations for better generalization.

## 6. Conclusion

To sum up, this 2-sample MR study found *Intestinimas.id.2062, Bifidobacterium.id.436, LachnospiraceaeUCG010.id.11330, RikenellaceaeRC9gutgroup.id.11191, RuminococcaceaeUCG014.id.11371* has a causal relationship with COVID-19. Further randomized controlled trials are needed to clarify the protective effect of probiotics against COVID-19 and the underlying mechanisms. In addition, although the reverse MR analysis does not support a causal relationship between COVID-19 and gut microbiota, it cannot be ruled out that COVID-19 may affect gut microecology, which needs to be confirmed by further studies.

## Author contributions

**Conceptualization:** Siyu Tian, Wenhui Huang.

**Data curation:** Siyu Tian.

**Methodology:** Siyu Tian, Wenhui Huang.

**Writing – original draft:** Siyu Tian.

**Funding acquisition:** Wenhui Huang.

**Software:** Wenhui Huang.

**Writing – review & editing:** Wenhui Huang.

## Supplementary Material




